# Effect of Mass on the Dynamic Characteristics of Single- and Double-Layered Graphene-Based Nano Resonators

**DOI:** 10.3390/ma15165551

**Published:** 2022-08-12

**Authors:** Manisha Makwana, Ajay M. Patel, Ankit D. Oza, Chander Prakash, Lovi Raj Gupta, Nikolai Ivanovich Vatin, Saurav Dixit

**Affiliations:** 1 Department of Mechanical Engineering, A. D. Patel Institute of Technology, Vallabh Vidyanagar 388121, India; 2 Department of Mechatronics Engineering, G. H. Patel College of Engineering & Technology, Vallabh Vidyanagar 388120, India; 3 Department of Computer Sciences and Engineering, Institute of Advanced Research, The University for Innovation, Gandhinagar 382426, India; 4 School of Mechanical Engineering, Lovely Professional University, Phagwara 144411, India; 5 Division of Research and Development, Lovely Professional University, Phagwara 144411, India; 6 Peter the Great St. Petersburg Polytechnic University, 195251 Saint Petersburg, Russia; 7 Division of Research & Innovation, Uttaranchal University, Dehradun 248007, India

**Keywords:** zigzag, armchair, chiral, SLG, DLG, mass sensor, frequency

## Abstract

Graphene has been widely and extensively used in mass sensing applications. The present study focused on exploring the use of single-layer graphene (SLG) and double-layer graphene (DLG) as sensing devices. The dynamic analysis of SLG and DLG with different boundary conditions (BDs) and length was executed using the atomistic finite element method (AFEM). SLG and DLG sheets were modelled and considered as a space–frame structure similar to a 3D beam. Spring elements (Combin14) were used to identify the interlayer interactions between two graphene layers in the DLG sheet due to the van der Waals forces. Simulations were carried out to visualize the behavior of the SLG and DLG subjected to different BDs and when used as mass sensing devices. The variation in frequency was noted by changing the length and applied mass of the SLGs and DLGs. The quantity of the frequency was found to be highest in the armchair SLG (6, 6) for a 50 nm sheet length and lowest in the chiral SLG (16, 4) for a 20 nm sheet length in the bridged condition. When the mass was 0.1 Zg, the frequency for the zigzag SLG (20, 0) was higher in both cases. The results show that the length of the sheet and the various mass values have a significant impact on the dynamic properties. The present research will contribute to the ultra-high frequency nano-resonance applications.

## 1. Introduction

Graphene was discovered in 2004, and its field of research now deals with various aspects of graphene. Graphene possesses amazing features, such as high electrical and thermal conductivity, and this drives the research [1,2,3,4]. It is a suitable material for flexible electronics and nanomechanical systems due to its higher stiffness, reduced mass per unit area, strength, and improved electrical conductivity nature [5,6,7,8,9]. A one-atom-thick planar sheet of sp2-bonded carbon atoms densely packed in a two-dimensional honeycomb crystal lattice is referred to as SLG. DLG is a stack of graphene sheets separated by 0.34 nanometres. Nanorings are made up of distorted graphite sheets, and they are the basic structural constituent of these structures. As one of the most widely used methods for producing graphene sheets, electrochemical exfoliation was the first synthetic process. Graphene sheets are thin sheets of graphene [10,11,12,13]. Microbial detections and diagnosis devices are all examples of nanodevices with outstanding properties in engineering and medicine [14,15,16,17]. The theoretical modelling of nanostructured materials can be conducted in three ways: (a) atomistic modelling, (b) hybrid atomistic-continuum mechanics, and (c) continuum mechanics, in addition to experimental studies. Classic molecular dynamics (MD) is an example of the atomic modelling approach [18]. MD simulations for the free vibration of multiple SLGs with varied lengths were utilized to find the suitable values of the nonlocal parameter. The focus of this research is to investigate the variation in the frequency of SLGs and DLGs for different values of the mass and length of the sheets using an atomistic model and MD simulations [19]. The resonant frequencies of the graphene sheets, the effects of the sheet sizes, the boundary conditions, and the number of layers were investigated. The vibrations of DLGs with various BDs between two layers were also considered. The modelling of the SLGs and DLGs is conducted at the atomic level [20]. The carbon atoms of the graphene sheets are covalently linked together to form a hexagonal lattice. Individual atoms cannot move due to the external forces and extreme bonds. Graphene can be described as a space–frame structure by seeing the bonds as connecting load-carrying elements and the atoms as the joints of the connecting elements. The mechanical behavior of graphene can be studied using standard structural mechanics methods if it is considered as a space–frame structure [21,22,23,24,25,26,27]. MD is an efficient technique for modeling graphene’s complete mechanical performance. However, it has a significant computing cost, which could be very costly for large-scale problems, particularly in the case of vibration analysis of graphene single- and double-layer sheets. The present work focused on the vibration analysis of SLG and DLG using zeptogram mass. For both designs, the results show a decrease in resonance frequencies as the associated mass increases.

### Van Der Waal Interaction in Graphene

At distances greater than 1 nm, the VdW force is significant in the interactions of atoms and molecules with carbon nanostructures [28]. Lifshitz and Pitaevskii developed a comprehensive VdW force theoretical framework based on the resonance dielectric permittivity principle, which is based on quantum statistical physics [29]. The intermolecular and atomic interaction with a microbody’s flat surface is described by this theory.

**Figure 1:**
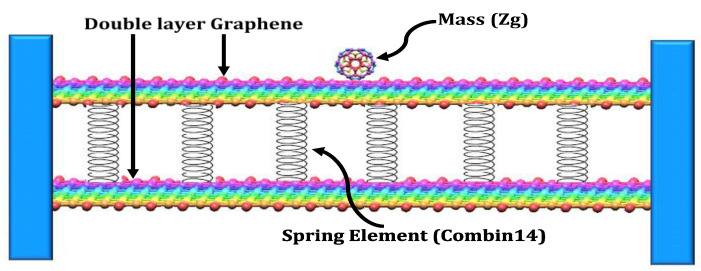
Double-layer graphene with spring element (Combin 14) and zeptogram mass attached in centre of sheet.

**Figure 2:**
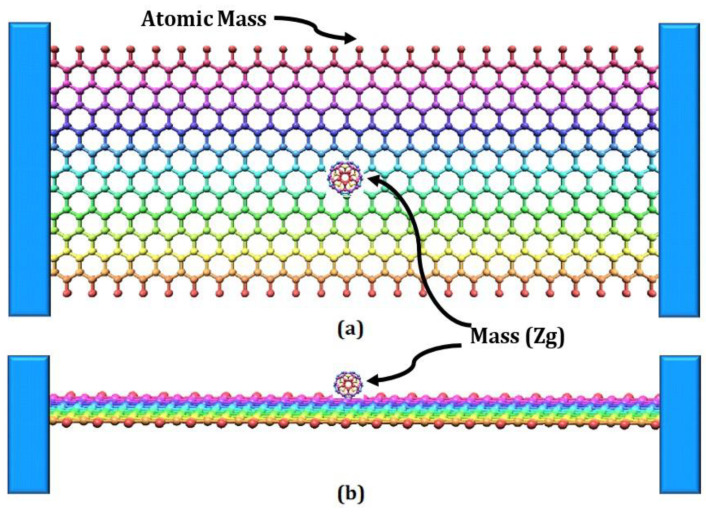
FEM model of bridged SLG with mass attached at centre. (**a**) SLG Top View with mass attached (**b**) SLG Front View Mass attached.

Figure 1 shows two layers of the double-layer graphene sheet; the spring element Combin 14 is used to represent the weak VdW force and the atomic mass at the node. The FEM model of the DLG is indicated in Figure 2.

## 2. Interatomic Modelling of SLG and DLG

The beam elements in the atomistic model accurately represent the covalent interactions between the carbon atoms. The elastic characteristics of the beam can be calculated by establishing links between the molecular and structural potential energy [30].

At low stresses, the total steric potential energy of a graphene can be calculated by adding the energies owing to the valence of the bonded contacts or the connected and non-bonded interactions [31].

In the present study, potential energy has been used to analyse linear nanospring stiffness by applying the finite element method. The sum of the forces exerted by the electrons and the electrostatic forces exerted between the positively charged nuclei equals the overall force exerted on each atomic nucleus.

(1) $$U=\sum U_{r}+\sum U_{\theta }+\sum U_{\phi }+\sum U_{\omega }+\sum U_{vdw}$$

where *U_r_* is the bond stretch interaction energy, *U_θ_* is the bending (bond angle variation energy), *U_ϕ_* is the dihedral angle torsion energy, *U_ω_* is the out-of-plane torsion energy, and *U_vdw_* is the non-bonded van der Waals interaction energy.

(2) $$U_{r}=\frac{1}{2}k_{r}{\left(r-r_{0}\right)}^{2}=\frac{1}{2}k_{r}{\left(\Delta r\right)}^{2}$$

(3) $$U_{\theta }=\frac{1}{2}k_{\theta }{\left(\theta -\theta_{0}\right)}^{2}=\frac{1}{2}k_{\theta }{\left(\Delta \theta \right)}^{2}$$

(4) $$U_{\tau }=U_{\phi }+U_{\omega }=\frac{1}{2}k_{\tau }{\left(\Delta \phi \right)}^{2}$$

where *k_r_*, *k_θ_*, and *k_τ_* are the bond stretching, bond bending, and torsional resistance force constants, respectively, while Δ*r*, Δ*θ* and Δ*ϕ* represent the bond stretching increment, the bond angle variation, and the angle variation of the bond twisting, respectively. The second derivatives of the potential energy terms in Equations (2)–(4) with respect to bond length, bond angle, and twisting bond angle variations produce the spring stiffness coefficients *k_r_*, *k_θ_*, and *k_τ_* according to Castigliano’s theorem.

The elements representing the bond are assumed to be elastic beams with Young’s modulus *E*, length *L*, cross-sectional area *A*, and moment of inertia *I*.

The strain energy under pure tension N is given by

(5) $$U_{A}=\frac{1}{2}\int_{0}^{l}\frac{N^{2}}{EA}dl=\frac{1}{2}\frac{N^{2}L}{EA}=\frac{EA}{L}{\left(\Delta L\right)}^{2}$$

The strain energy of the beam element under the pure bending moment *M* can be expressed as:

(6) $$U_{M}=\frac{1}{2}\int_{0}^{l}\frac{M^{2}}{EI}dl=\frac{2EI}{L}{\left(\alpha \right)}^{2}=\frac{EI}{L}{\left(2\alpha \right)}^{2}$$

where *α* is the rotational angle of the beam ends.

Similarly, the strain energy of the beam element under pure twisting moment *T* is given by

(7) $$U_{T}=\frac{1}{2}\int_{0}^{l}\frac{T^{2}}{GJ}dl=\frac{1}{2}\frac{T^{2}L}{GJ}=\frac{1}{2}\frac{GJ}{L}{\left(\Delta \beta \right)}^{2}$$

where *J*, *G*, and Δβ are the polar moment of inertia, the shear modulus, and the relative rotations of the beam ends, respectively [20].

(8) $$\frac{EA}{L}=K_{r},\;\frac{EI}{L}=K_{\theta },\;\frac{GJ}{L}=K_{\tau }$$

The elastic properties of the beam element are given as [21]

(9) $$d=\sqrt{\frac{k_{\theta }}{k_{r}}},\;E=\frac{k_{r}^{2}L}{4\pi k_{\theta }},G=\frac{k_{r}^{2}k_{\tau }^{L}}{8\pi k_{\theta }^{2}},$$

where *d*, *L*, *E*, and *G* represent the diameter, length, Young’s modulus, and shear modulus of the beam element.

The stiffness *K_s_* of the special spring is defined by the following equation:

(10) $$K_{s}={\left(\frac{1}{a_{c-c}\cos{\left(60\right)}^{0}}\right)}^{2}k_{\theta }$$

For the Finite Element Model of DLG, as indicated in Figure 2, the DLGs are modeled considering the weak van der Waals force of attraction between the upper and lower layers as a spring element. The van der Waals force field between the interfacial layers is represented by the spring element Combin 14.

The spring stiffness coefficients of Equations (2)–(4) are taken to be equal to *k_r_* = 6.52 × 10^−7^ N nm^−1^, *k_θ_* = 8.76 × 10^−10^ N nm rad^−2^, and *k_t_* = 2.78 × 10^−10^ N nm rad^−2^ [32,33].

### 2.1. Single-Layer Graphene Sheets Analytical Approach

**Figure 3:**
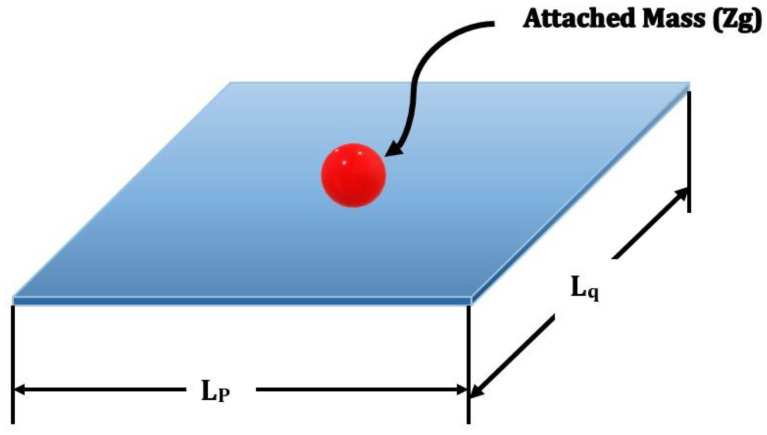
A single-walled GS with an attached mass is shown in a schematic diagram.

We analyse the dynamic behaviour of an SLG with an attached concentrated mass (Zg) at an arbitrary place using nonlocal continuum mechanics, as shown in Figure 3. The graphene sheet’s origin is located in one of its corners in the mid-plane [34,35,36,37,38,39,40,41,42,43,44,45,46,47,48,49].

The *x*- and *y*-axes are aligned with the SLG’s length Lp and width Lq, respectively, and the *z*-axis is aligned with the SLG’s thickness *h* [50].

The two-dimensional nonlocal constitutive equations of the SLGs are written using nonlocal elasticity theory, as follows:

(11) $$\begin{array}{l} \sigma_{xx}-{\left(e_{0a}\right)}^{2}\left(\frac{\sigma^{2}xx}{\sigma x^{2}}+\frac{\sigma^{2}xx}{\sigma y^{2}}\right)=\frac{E}{1-{\left(v\right)}^{2}}\left(\varepsilon_{xx}+v\varepsilon_{xx}\right)\\ \sigma_{yy}-{\left(e_{0a}\right)}^{2}\left(\frac{\sigma^{2}yy}{\sigma x^{2}}+\frac{\sigma^{2}yy}{\sigma y^{2}}\right)=\frac{E}{1-{\left(v\right)}^{2}}\left(\varepsilon_{yy}+v\varepsilon_{xx}\right)\\ \tau_{xy}-{\left(e_{0a}\right)}^{2}\left(\frac{\sigma^{2}xy}{\sigma x^{2}}+\frac{\sigma^{2}xy}{\sigma y^{2}}\right)=G\gamma_{xy} \end{array}$$

where *E*, *G*, and *ν* are the elastic modulus, the shear modulus, and the Poisson’s ratio of the GSs, respectively. The internal characteristic length a is the distance between two atoms in a C-C bond, which is 0.142 nm.

(12) $$\begin{array}{l} M_{xx}=\int_{\frac{-h}{2}}^{\frac{h}{2}}z\sigma_{xx}dz\\ M_{yy}=\int_{\frac{-h}{2}}^{\frac{h}{2}}z\sigma_{yy}dz\\ M_{xy}=\int_{\frac{-h}{2}}^{\frac{h}{2}}z\tau_{xy}dz \end{array}$$

The link between the strain and the displacement fields is represented as being when the middle surface displacements in the *x* and *y* directions are ignored.

(13) $$\begin{array}{l} \varepsilon_{xx}=-z\frac{\partial^{2}w}{\partial x^{2}}\\ \varepsilon_{xx}=-z\frac{\partial^{2}w}{\partial y^{2}}\\ \gamma_{xx}=-2z\frac{\partial^{2}w}{\partial x\partial y} \end{array}$$

where *w* is the displacement along the GS’ thickness. We obtain Equation (15) by substituting Equation (13) into Equation (14)

(14) $$\begin{array}{l} M_{xx}-{\left(e_{0a}\right)}^{2}\left(\frac{\partial^{2}Mxx}{\partial x^{2}}+\frac{\partial^{2}Mxx}{\partial y^{2}}\right)=-D\left(\frac{\partial^{2}\omega }{\partial x^{2}}+v\frac{\partial^{2}\omega }{\partial y^{2}}\right)\\ M_{yy}-{\left(e_{0a}\right)}^{2}\left(\frac{\partial^{2}Myy}{\partial x^{2}}+\frac{\partial^{2}Myy}{\partial y^{2}}\right)=-D\left(\frac{\partial^{2}\omega }{\partial y^{2}}+v\frac{\partial^{2}\omega }{\partial x^{2}}\right)\\ M_{xy}-{\left(e_{0a}\right)}^{2}\left(\frac{\partial^{2}Mxy}{\partial x^{2}}+\frac{\partial^{2}Mxy}{\partial y^{2}}\right)=-D(1-v)\frac{\partial^{2}\omega }{\partial y\partial x} \end{array}$$

where *D* is the flexural rigidity of SLGs, expressed as

$$D=-z\frac{Eh^{3}}{12(1-v^{2})}$$

The governing equation for the flexural vibration of SLGs carrying a nanoparticle can be given as

(15) $$\begin{array}{l} \left(\frac{\partial^{2}Mxx}{\partial x^{2}}+2\frac{\partial^{2}Mxy}{\partial y\partial x}+\frac{\partial^{2}Mxx}{\partial y^{2}}\right)=\left[\rho h+m_{c}\delta (x-x_{0})\delta (y-y_{0})\right]\frac{\partial^{2}\omega }{\partial t^{2}} \end{array}$$

where *m_c_* is the mass of nanoparticles connected at point (*x*_0_, *y*_0_) and is the Dirac delta function indicated by

(16) $$\begin{array}{l} \delta \left(x\right)=\{{}_{0,}^{+\infty ,}\\ x=0\\ x\neq 0 \end{array}$$

Substituting Equation (17) into Equation (18), the governing equation can be written in terms of *w* as

(17) $$D\left(\frac{\partial^{4}w}{\partial x^{4}}+2\frac{\partial^{4}w}{\partial x^{2}\partial y^{2}}+\frac{\partial^{4}w}{\partial y^{4}}\right)+\left[1-{\left(e_{0}a\right)}^{2}\left(\frac{\partial^{2}w}{\partial x^{2}}+\frac{\partial^{2}w}{\partial y^{2}}\right)\right]\left[\rho h+m_{c}\delta (x-\xi )\delta (x-\eta )\right]\frac{\partial^{2}\omega }{\partial t^{2}}=0$$

The harmonic solution of Equation (19) can be expressed as

(18) $$W(x,\;y,\;t)=Y(x,\;y)e^{iwt}$$

where *Y* (*x*, *y*) is the shape function of deflection, and *ω* is the resonant frequency of the SLGs.

Substituting Equation (20) into Equation (19), we obtain

(19) $$D\left(\frac{\partial^{4}Y}{\partial x^{4}}+2\frac{\partial^{4}Y}{\partial x^{2}\partial y^{2}}+\frac{\partial^{4}Y}{\partial y^{4}}\right)+\frac{\omega^{2}}{D}\left[1-{\left(e_{0}a\right)}^{2}\left(\frac{\partial^{2}Y}{\partial x^{2}}+\frac{\partial^{2}Y}{\partial y^{2}}\right)\right]\left[\rho h+m_{c}\delta (x-\xi )\delta (x-\eta )\right]\frac{\partial^{2}Y}{\partial x^{2}}=0$$

Note that the boundary conditions of the SLGs with simply supported edges are

(20) $$\begin{array}{l} \omega =0\\ \frac{\partial^{2}w}{\partial x^{2}}=0\\ \frac{\partial^{2}w}{\partial y^{2}}=0 \end{array}$$

On *x* = 0, $L_{p}$ and *y* = 0 $L_{q}$.

Therefore, the shape function (*Y*) in Equation (20) can be expressed as

(21) $$Y(x,y)=A_{\mathrm{mn}}\;\sin\frac{m\pi x}{L_{p}}\;\sin\frac{n\pi y}{L_{q}}$$

where $A_{\mathrm{mn}}$ denotes the oscillation’s vibration amplitude, and *m* and *n* denote the mode numbers in the periodic directions. After some optimizations, we obtain the following frequency equation by substituting the shape function of Equation (23) into Equation (21), multiplying both sides of Equation (17) by sin*mx*/*L_p_* sin*ny*/*L_q_*, and integrating over the entire region with respect to *x* and *y*, with the limits *x* = 0 to *x* = *L_p_* and *y* = 0 to *y* = *L_q_*.

(22) $$\begin{array}{l} \int_{0}^{b}\int_{0}^{a}A_{mn}D\prod^{4}{\left(\frac{m^{2}}{L^{2}p}+\frac{m^{2}}{L^{2}q}\right)}^{2}\sin^{2}\frac{m\prod x}{Lp}\sin^{2}\frac{n\prod y}{Lq}d_{x}d_{y}\\ -\omega^{2}A_{mn}\left[1+{\left(e_{0}a\right)}^{2}\prod^{2}{\left(\frac{m^{2}}{L^{2}p}+\frac{m^{2}}{L^{2}q}\right)}^{2}\right]\left[\rho h+m_{c}\delta (x-\xi )\delta (x-\eta )\right]\sin^{2}\frac{m\prod x}{Lp}\sin^{2}\frac{n\prod y}{Lq}d_{x}d_{y}=0 \end{array}$$

The required resonant frequencies corresponding to a given form function are all roots of the above equation. For the non-trivial solution, the *A_mn_* coefficient should be zero. As a result, the resonant frequency of a mass sensor may be calculated.

(23) $$\omega_{n}{}^{2}=\frac{D\prod^{4}{\left(\frac{m^{2}}{L^{2}p}+\frac{m^{2}}{L^{2}q}\right)}^{2}}{\left[1+{\left(e_{0}a\right)}^{2}\prod^{2}{\left(\frac{m^{2}}{L^{2}p}+\frac{m^{2}}{L^{2}q}\right)}^{2}\right]\left(\rho h+\frac{4mc}{LpLq}\right)\sin^{2}m\prod \zeta \sin^{2}n\prod \eta }$$

The resonance frequency of an SLG with connected nanoparticles may also be calculated using classical elasticity theory when the nonlocal parameter ($e_{0a}$) is considered to be zero. Graphene is an enormously robust nanomaterial, with a Young’s module near to 1 TPa [51,52,53].

### 2.2. Validation of the Model

We obtained an analytical technique from Natsuki et al. [50] to compare with the FEA model with the objective of validating the existing model results. As shown in Table 1, the analytical and FEA results are in close proximity. The following material attributes were considered by the authors when validating the SLG model: E = 3792.47739 nN/nm^2^, ν = 0.17, and ρ = 8362.714 kg/m^3^.

**Figure 4:**
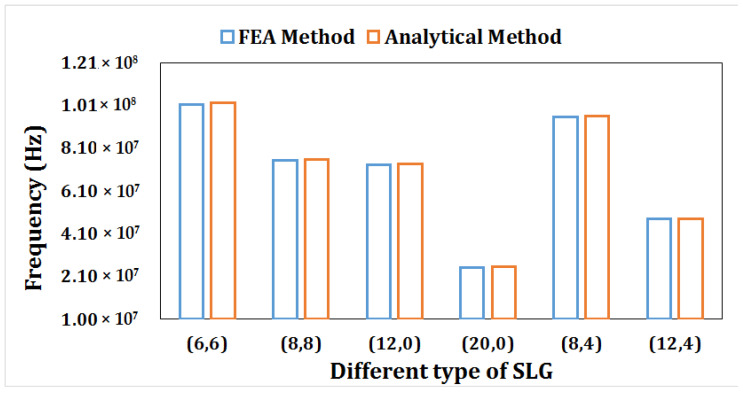
Compression of first mode of frequency of current FEA model and analytical model of Natsuki [50].

The results are presented in Figure 4. The frequency variation between the analytical model and the simulated FEM proximity has been identified. However, the current model for the application as a mass sensing device is reflected in the results of the FEA technique and analytical results.

## 3. Results and Discussion

The SLG and DLG frame structures were modelled. Nine different varieties of SLGs and DLGs were evaluated for analysis, including three different types of armchairs (8, 8), (6, 6), (10, 10); zigzag (20, 0); (16, 0), (12, 0); and chiral (16, 4), (12, 4), (8, 4). The influence of the diverse boundary conditions, such as cantilever and bridged, on the mass sensing ability of the DLG- and SLG-based mass sensors was investigated.

Table 2 shows the fundamental frequency of several types of cantilever DLGs with various mass additions at the tip of sheet. Table 3 demonstrates the frequency of several types of bridged DLGs with various mass additions in the sheet’s centre point. When the mass is 0.1 Zg, the frequency for the zigzag DLG (20, 0) is higher in both cases. Furthermore, when the associated mass on the tip and centre of the DLG increases, the fundamental frequency decreases. Table 4 shows the fundamental frequency for several cantilever SLGs with various mass additions at the sheet’s tip. Table 5 demonstrates the frequency of several types of bridged SLGs with various mass additions at the centre of the sheet. When the mass is 0.1 Zg, the frequency for the zigzag SLG (20, 0) is higher in both cases. Moreover, as the associated mass on the tip and centre of SLGs increases, the fundamental frequency decreases.

**Figure 5:**
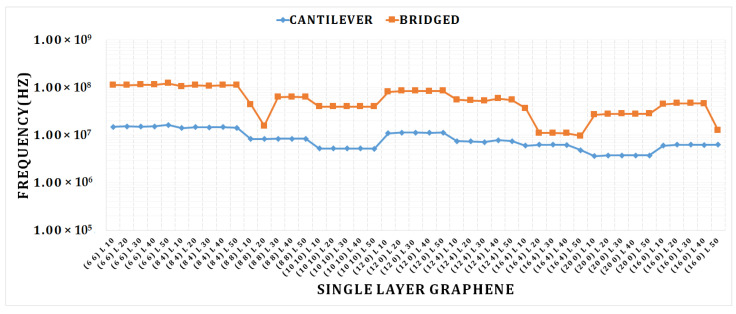
Cantilever and bridged SLGs; graph for different length and chirality vs. frequency.

**Figure 6:**
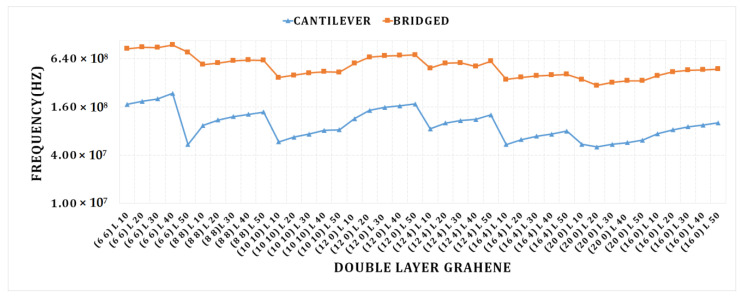
Cantilever and bridged DLGs; graph for different length and chirality vs. frequency.

The frequency of the cantilever SLG fluctuates with length and chirality, as shown in Figure 5. The quantity of the frequency was found to be larger in the zigzag SLG (6, 6) for a sheet length of 50 nm and lower in the armchair SLG (20 0) for a sheet length of 50 nm in the cantilever condition. The quantity of frequency fluctuates for the different lengths and chirality of the bridged SLGs; this is shown in Figure 6. The quantity of frequency was found to be highest in the armchair SLG (6, 6) for a 50 nm sheet length and lowest in the chiral SLG (16, 4) for a 20 nm sheet length in the bridged condition. The graph clearly shows that as chirality increases, the frequency drops. In a smaller graphene, a higher frequency can be attained. In the bridged state of single-layer graphene, there is a huge variation in frequency. It shows that the bridged condition has a higher frequency.

The graph in Figure 6 shows that a considerable fluctuation in frequency is attained at different lengths of the double-layered graphene sheet. As the chirality of a frequency grows, its value eventually falls. With a smaller graphene sheet, the armchair cantilever SLG (6, 6), and a sheet length of 40, we obtain the highest frequency value. Its frequency begins to decrease as its chirality increases. The greatest frequency is attained for the smallest cantilever armchair DLG (6, 6). DLG (20, 0) achieves the lowest frequency value at a length of 20 nm.

The bridged armchair DLG (6, 6) with a length of 50 nm achieves the highest frequency. We can see that as the chirality increases, the value of the frequency drops in the bridged-conditioned double-layer graphene as well. The minimum frequency for DLG (20, 0) at length 20 nm is obtained under the bridged state.

**Figure 7:**
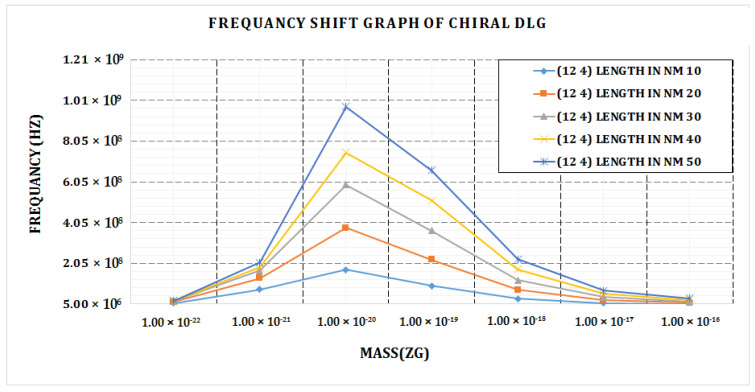
Frequency shift vs. attached mass graph of bridged chiral DLG (12, 4).

We discovered the highest frequency value for bridged chiral DLG (12, 4) at length 50 nm and mass attached 1 × 10^−20^ using the frequency shift graph given in Figure 7. For the shortest length of 10 nm of bridged DLG, we obtain the lowest frequency shift (12, 4). As the length of the graph grows, so does the frequency shift.

**Figure 8:**
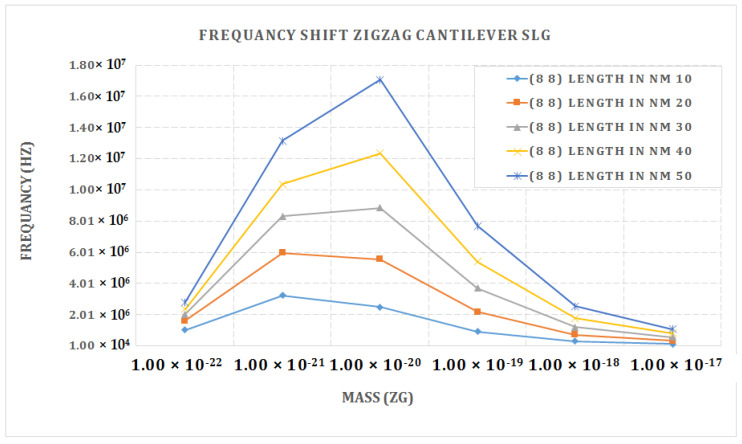
Frequency shift vs. attached mass (Zg) graph of cantilever armchair SLG (8, 8).

Figure 8 shows the frequency shift graph of the armchair single-layer cantilever graphene SLG (8, 8). The maximum value frequency was attained for the longest length of 50 nm and the shortest length of 10 nm of a double-layer graphene sheet. The mass value of 1 × 10^−20^ was found to give the highest frequency.

**Figure 9:**
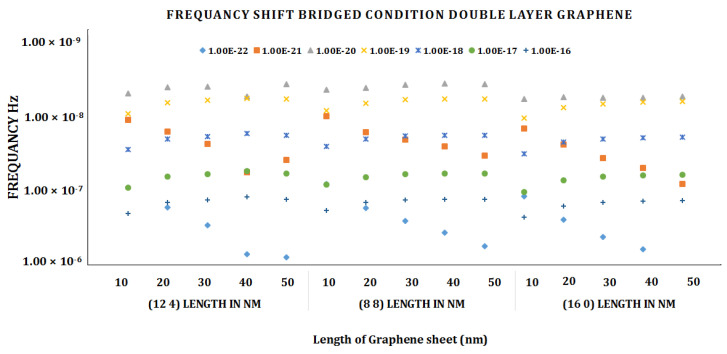
Frequency shift vs. different lengths of graphene sheet (nm) graph for chiral, armchair, and zigzag bridged DLG.

Figure 9 depicts the frequency shift caused by mass change at several positions of the bridging SLG (8, 8), (16, 0), (18, 0), and (12, 4). It can be shown that at lower mass values, there are far fewer frequency shifts in the SLG, which significantly rises as the attached mass grows. For an attached mass of more than 0.1 zg, the amount of frequency shift is observed to be larger in SLG (8, 8) at a length of 50 nm. This clearly indicates that the frequency fluctuation is much less at lower mass values. It suggests that SLGs with a mass sensitivity of 0.1 zg can be impacted by changes in the sheet length.

**Figure 10:**
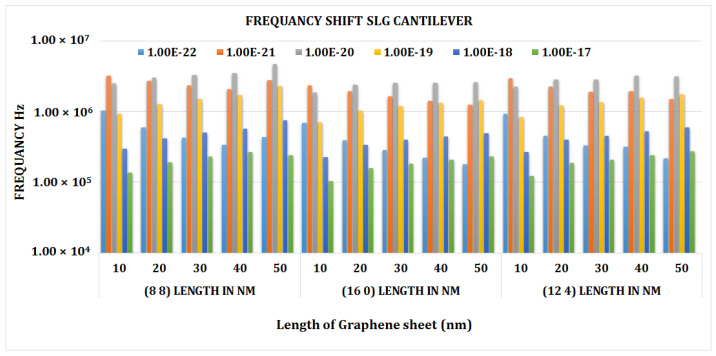
Frequency shift vs. length of graphene sheet for chiral, armchair, and zigzag cantilever SLG.

Figure 10 depicts the frequency shift caused by mass fluctuation at different cantilever SLGs (8, 8), (16, 0), and (12, 4). It can be shown that at lower mass values, there will be far fewer frequency shifts in the SLG, which significantly rises as the attached mass increases. For an attached mass of more than 0.1 zg, the amount of frequency shift is found to be larger in the armchair SLG (8, 8) at a length of 50 nm, and the minimum frequency is found in the chiral SLG (12, 4). This clearly indicates that the frequency fluctuation is much less at lower mass and chirality values. It suggests that SLGs with a mass sensitivity of 0.1 zg can be impacted by changes in sheet length.

**Figure 11:**
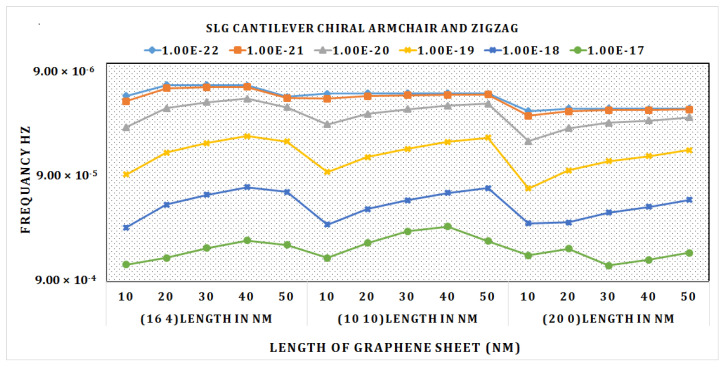
Graph of frequency (Hz) vs. different lengths of chiral, armchair, and zigzag cantilever DLG.

According to the graph in Figure 11, at a length of 20 nm the frequency was highest in the cantilever chiral SLG (16, 4). As the length of the sheet grows longer, it steadily diminishes. As demonstrated in the graph, the zigzag SLG (20, 0) has the lowest frequency at a length of 20 nm.

The graph shows that when the chirality grows, the frequency drops progressively. We can determine from this graph that the maximum frequency can be reached with reduced chirality and length. The frequency drops as the mass on the sheet increases.

**Figure 12:**
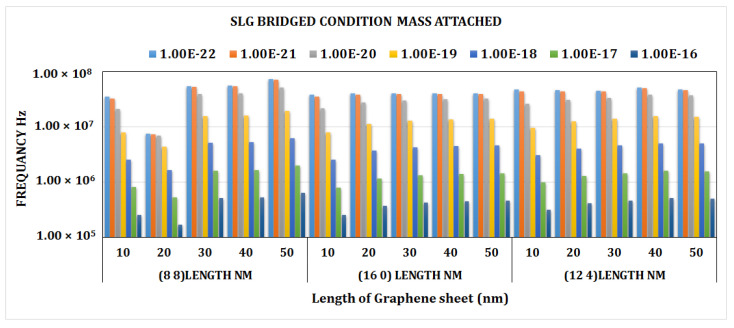
Frequency shift vs. length of graphene sheet of chiral, armchair, and zigzag cantilever SLG.

Figure 12 illustrates the frequency shift due to mass fluctuation at various lengths of SLG (8, 8), (16, 0), (18, 0), and (12, 4). It can be shown that at lower mass values, there were much smaller frequency shifts in the SLG, which significantly rises as the attached mass grows. For an attached mass of greater than 0.1 zg, the highest amount of frequency shift was discovered in SLG (8, 8), at a length of 50 nm. This clearly indicates that the frequency fluctuation is much less at lower mass values. It suggests that SLGs with a mass sensitivity of 0.1 zg can be impacted by changes in the sheet length.

**Figure 13:**
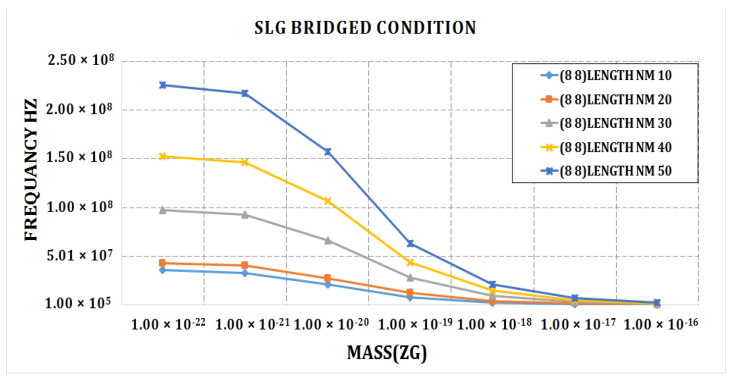
Frequency vs. mass graph for bridged armchair SLG (8, 8).

The graph shows in Figure 13. that in single-layer graphene at maximum length, maximum frequency may be reached. The graph clearly demonstrates that as the length of the sheet rises, the frequency increases as well, with the highest frequency at 50 nm. As the length lowers and the mass rises, the frequency decreases gradually. As a result, the frequency variation will be much less with higher mass.

**Figure 14:**
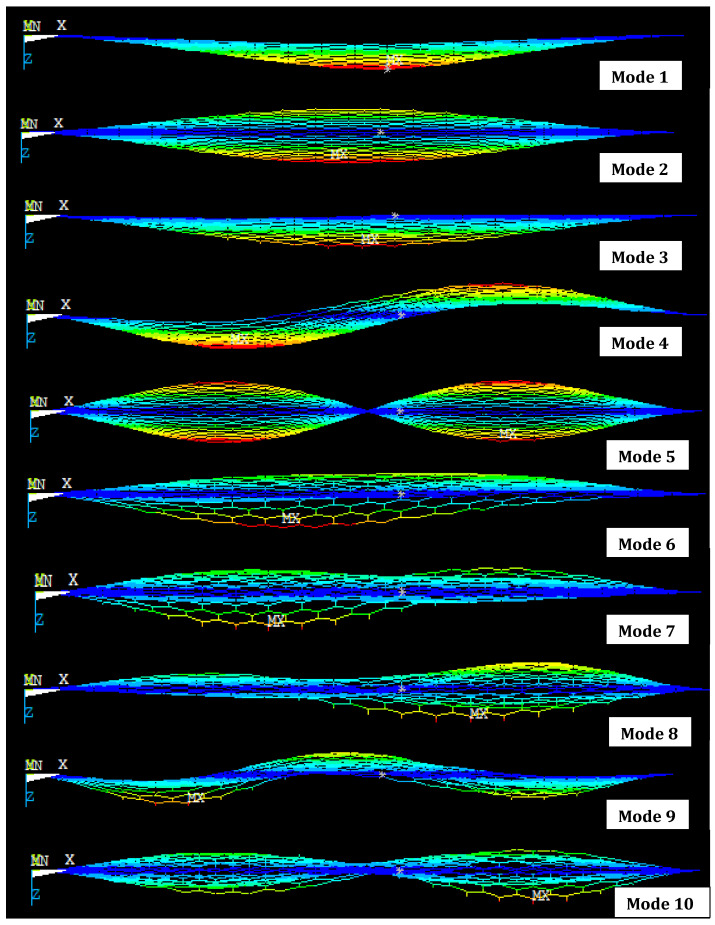
First ten mode shapes of bridged SLG (16, 4) with length 50 nm (front view of mode shape).

Figure 14 shows the first ten mode shapes of vibration for the bridged SLG (16, 4) with the length of 50 nm. The vibration of the bridged chiral SLG (16, 4) rises from the second mode, as can be seen from the mode shape above. Mode 5 shows a half-sine-wave shape of response.

FEA results show areas of high stress with red color, lower than that with green and yellow color and lowest stress with blue. MN is stress at the fixed end and MX stress point at the span of layer. 

**Figure 15:**
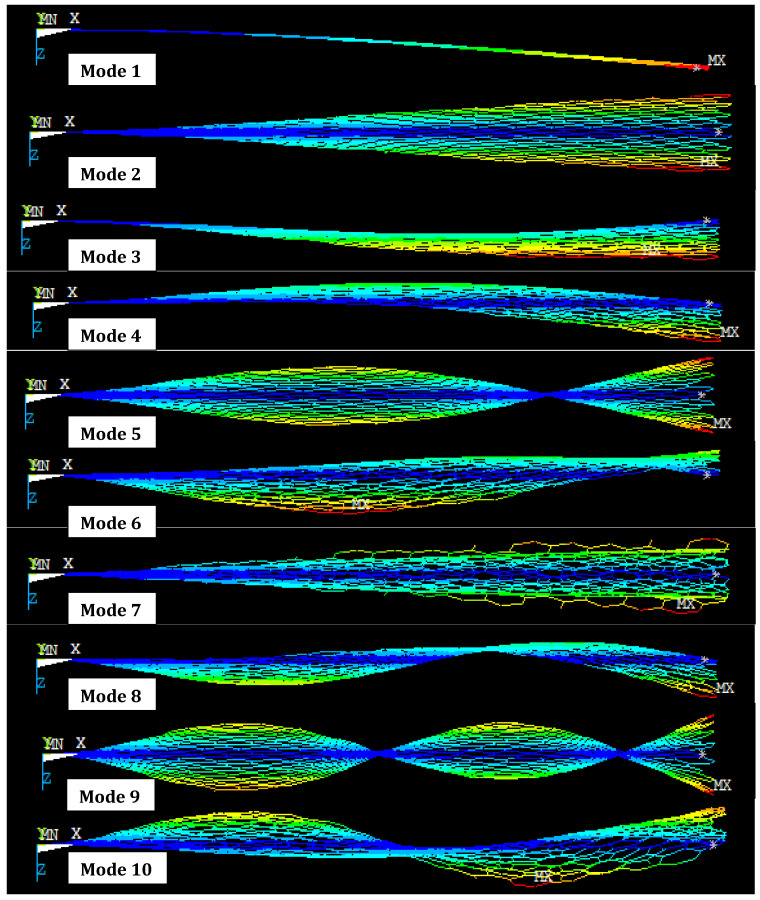
Different mode shapes of cantilever SLG (16, 4) with length 50 nm (front view of mode shape).

As shown in Figure 15. The vibrations increase with the second mode of the cantilever single-layer graphene chiral graphene (16, 4) and continue to increase until the last mode of vibration. A nonlinear pattern is observed for the different mode shapes.

**Figure 16:**
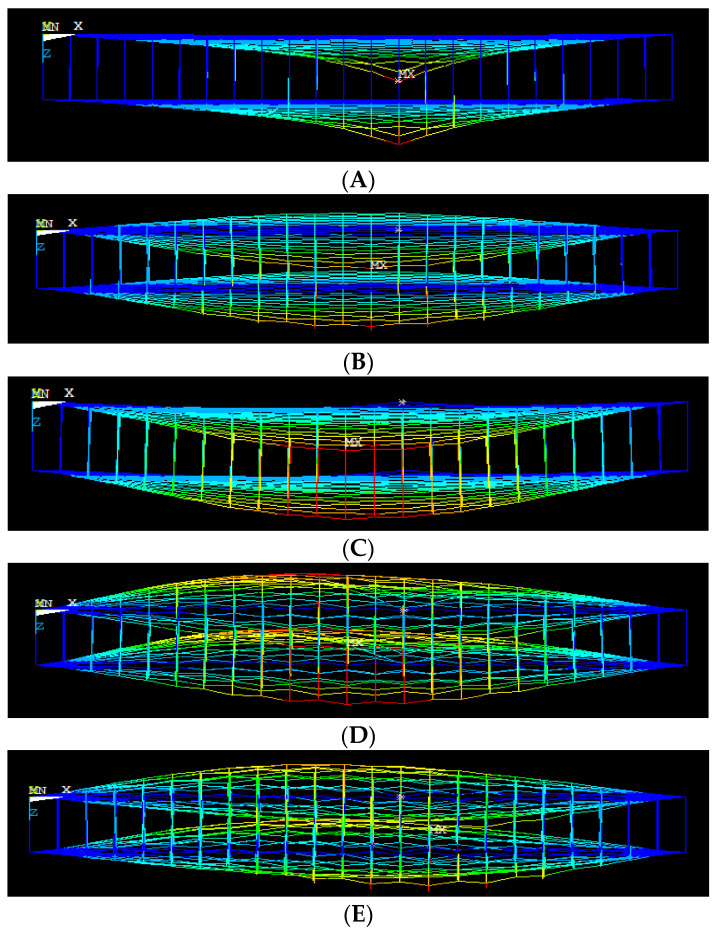
(**A**–**E**) Different mode shapes of bridged DLG (20, 0) with length 50 nm (front view of mode shape).

Figure 16 shows the first ten mode shapes of bridged DLG (20, 0) with a length of 50 nm. The variation in the vibration of the sheet from the second mode can be seen from the mode shape of the bridged DLG (20, 0). The last mode shows a non-coaxial vibration of response.

**Figure 17:**
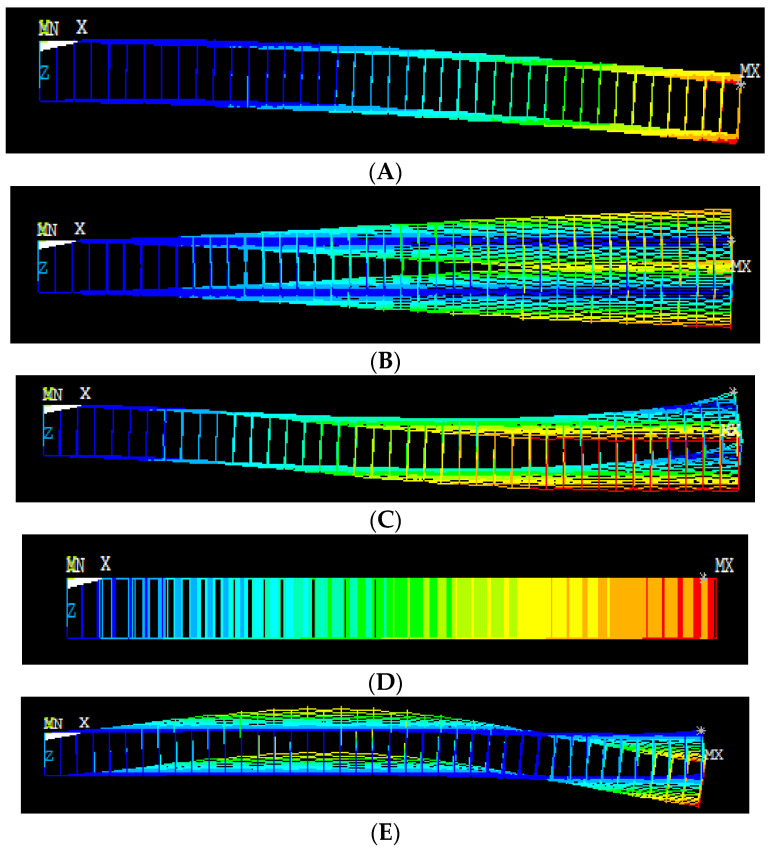

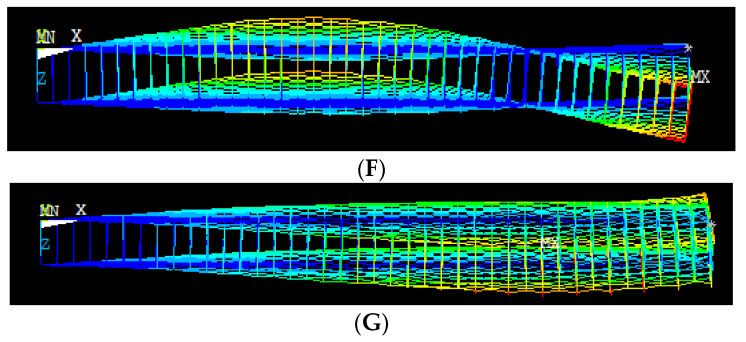
(**A**–**G**) Different mode shapes of cantilever DLG (20, 0) with length 50 nm (front view of mode shape).

The variation in the vibration of the sheet as shown in Figure 17. starts from the second mode, as shown in the mode shape of the cantilever DLG (20, 0).

## 4. Conclusions

To investigate the vibrational characteristics of the cantilever and bridged double-layer zigzag, armchair, and chiral graphene sheets, an atomistic FEA model for the van der Waal interaction between the upper and lower graphene sheets and the concentrated masses was prepared.

The frequency shift graph of SLG and DLG for the bridging and cantilever conditions concludes that as the length of the sheet increases, so does its frequency, and as the mass of the sheet increases, the frequency also increases. The effect of different lengths of single- and double-layer graphene sheets on frequency was studied using an atomistic modelling technique. It was discovered that the longer the graphene sheet, the higher the frequency. The impacts of mass attached to the tip of the single-layer graphene and the double-layer graphene for the cantilever conditions, as well as to the centre of a bridging graphene sheet, were investigated. In both cases, maximum frequency was reached for mass values greater than 1 zg. The vibrations began with the second mode shape as the SLG and DLG vibrate in different planes, as observed from the mode shapes of the SLGs and DLGs for the cantilever and bridged states. In the bridged DLG (6, 6) with a 1.00 × 10^−16^ mass attached at the centre of the sheet, the maximum frequency achieved was 7.76 × 10^6^, and in bridged SLG (6, 6) with a similar mass attached, the highest frequency obtained was 7.50 × 10^5^. The results show that the graphene sheet (6, 6) with a length of 50 nm achieves the maximum frequency for both the bridged SLG and the DLG. This research will help in future applications of graphene in advanced nano-resonator applications.

## Figures and Tables

**Table 1:** Results comparison of FEA method and analytical method.

Graphene Type	Single Layer	Length of Graphene	FEA Method Frequency (Hz)	Analytical Method [53] Frequency (Hz)
Armchair	(6, 6)	10	1.0136 × 10^8^	1.0235 × 10^8^
	(8, 8)	20	7.5488 × 10^7^	7.5887 × 10^7^
Zigzag	(12, 0)	10	7.3185 × 10^7^	7.3574 × 10^7^
	(20, 0)	20	2.5121 × 10^7^	2.5489 × 10^7^
Chiral	(8, 4)	10	9.5844 × 10^7^	9.5987 × 10^7^
	(12, 4)	20	4.7985 × 10^7^	4.8042 × 10^7^

**Table 2:** Cantilever zigzag and armchair DLG with mass addition at tip of sheet.

Length of Sheet (Nm)	Mass (gm)						
	1.00 × 10^−22^	1.00 × 10^−21^	1.00 × 10^−20^	1.00 × 10^−19^	1.00 × 10^−18^	1.00 × 10^−17^	1.00 × 10^−16^
**DLG Zigzag (6, 6)**							
10	1.71 × 10^8^	1.62 × 10^8^	1.14 × 10^8^	4.57 × 10^7^	1.49 × 10^7^	4.73 × 10^6^	1.50 × 10^6^
20	1.97 × 10^8^	1.92 × 10^8^	1.52 × 10^8^	6.82 × 10^7^	2.26 × 10^7^	7.19 × 10^6^	2.28 × 10^6^
30	2.14 × 10^8^	2.10 × 10^8^	1.75 × 10^8^	8.22 × 10^7^	2.74 × 10^7^	8.73 × 10^6^	2.76 × 10^6^
40	2.26 × 10^8^	2.23 × 10^8^	1.94 × 10^8^	9.57 × 10^7^	3.22 × 10^7^	1.02 × 10^7^	3.24 × 10^6^
50	2.36 × 10^8^	2.33 × 10^8^	2.03 × 10^8^	9.63 × 10^7^	3.20 × 10^7^	1.02 × 10^7^	3.21 × 10^6^
**DLG Zigzag (8, 8)**							
10	9.35 × 10^7^	8.99 × 10^7^	6.75 × 10^7^	2.89 × 10^7^	9.55 × 10^6^	3.03 × 10^6^	9.60 × 10^5^
20	1.10 × 10^8^	1.08 × 10^8^	9.00 × 10^7^	4.41 × 10^7^	1.50 × 10^7^	4.77 × 10^6^	1.51 × 10^6^
30	1.21 × 10^8^	1.19 × 10^8^	1.04 × 10^8^	5.50 × 10^7^	1.90 × 10^7^	6.06 × 10^6^	1.92 × 10^6^
40	1.30 × 10^8^	1.29 × 10^8^	1.15 × 10^8^	6.32 × 10^7^	2.20 × 10^7^	7.01 × 10^6^	2.22 × 10^6^
50	1.38 × 10^8^	1.37 × 10^8^	1.24 × 10^8^	6.93 × 10^7^	2.41 × 10^7^	7.68 × 10^6^	2.43 × 10^6^
**DLG Zigzag (10, 10)**							
10	5.95 × 10^7^	5.76 × 10^7^	4.50 × 10^7^	2.02 × 10^7^	6.72 × 10^6^	2.14 × 10^6^	6.76 × 10^5^
20	6.71 × 10^7^	6.60 × 10^7^	5.70 × 10^7^	2.99 × 10^7^	1.04 × 10^7^	3.32 × 10^6^	1.05 × 10^6^
30	7.59 × 10^7^	7.50 × 10^7^	6.73 × 10^7^	3.84 × 10^7^	1.37 × 10^7^	4.39 × 10^6^	1.39 × 10^6^
40	8.17 × 10^7^	8.10 × 10^7^	7.43 × 10^7^	4.48 × 10^7^	1.63 × 10^7^	5.22 × 10^6^	1.65 × 10^6^
50	8.66 × 10^7^	8.60 × 10^7^	8.00 × 10^7^	4.99 × 10^7^	1.83 × 10^7^	5.88 × 10^6^	1.86 × 10^6^
**DLG Armchair (12, 0)**							
10	1.14 × 10^8^	1.11 × 10^8^	9.29 × 10^7^	4.57 × 10^7^	1.55 × 10^7^	4.96 × 10^6^	1.57 × 10^6^
20	1.45 × 10^8^	1.42 × 10^8^	1.18 × 10^8^	5.69 × 10^7^	1.93 × 10^7^	6.14 × 10^6^	1.94 × 10^6^
30	1.58 × 10^8^	1.56 × 10^8^	1.33 × 10^8^	6.57 × 10^7^	2.22 × 10^7^	7.06 × 10^6^	2.24 × 10^6^
40	1.65 × 10^8^	1.63 × 10^8^	1.43 × 10^8^	7.21 × 10^7^	2.44 × 10^7^	7.77 × 10^6^	2.46 × 10^6^
50	1.74 × 10^8^	1.72 × 10^8^	1.53 × 10^8^	7.78 × 10^7^	2.62 × 10^7^	8.34 × 10^6^	2.64 × 10^6^
**DLG Armchair (16, 0)**							
10	7.39 × 10^7^	7.14 × 10^7^	5.52 × 10^7^	2.44 × 10^7^	8.11 × 10^6^	2.58 × 10^6^	8.16 × 10^5^
20	8.30 × 10^7^	8.15 × 10^7^	7.00 × 10^7^	3.63 × 10^7^	1.25 × 10^7^	4.01 × 10^6^	1.27 × 10^6^
30	9.04 × 10^7^	8.92 × 10^7^	7.92 × 10^7^	4.35 × 10^7^	1.53 × 10^7^	4.88 × 10^6^	1.54 × 10^6^
40	9.49 × 10^7^	9.39 × 10^7^	8.51 × 10^7^	4.90 × 10^7^	1.73 × 10^7^	5.55 × 10^6^	1.76 × 10^6^
50	1.01 × 10^8^	1.00 × 10^8^	9.22 × 10^7^	5.44 × 10^7^	1.93 × 10^7^	6.18 × 10^6^	1.96 × 10^6^
**DLG Armchair (20, 0)**							
10	5.46 × 10^7^	5.29 × 10^7^	4.18 × 10^7^	1.90 × 10^7^	6.35 × 10^6^	2.02 × 10^6^	6.39 × 10^5^
20	5.05 × 10^7^	4.97 × 10^7^	4.34 × 10^7^	2.34 × 10^7^	8.20 × 10^6^	2.62 × 10^6^	8.31 × 10^5^
30	5.46 × 10^7^	5.41 × 10^7^	4.95 × 10^7^	3.01 × 10^7^	1.11 × 10^7^	3.57 × 10^6^	1.13 × 10^6^
40	5.72 × 10^7^	5.67 × 10^7^	5.26 × 10^7^	3.30 × 10^7^	1.23 × 10^7^	3.95 × 10^6^	1.25 × 10^6^
50	6.14 × 10^7^	6.10 × 10^7^	5.72 × 10^7^	3.73 × 10^7^	1.40 × 10^7^	4.53 × 10^6^	1.43 × 10^6^

**Table 3:** Bridged DLG zigzag and armchair with mass addition at centre of sheet.

Length of Sheet (Nm)	Mass (gm)						
	1.00 × 10^−22^	1.00 × 10^−21^	1.00 × 10^−20^	1.00 × 10^−19^	1.00 × 10^−18^	1.00 × 10^−17^	1.00 × 10^−16^
**DLG Zigzag (6, 6)**							
10	6.81 × 10^8^	6.58 × 10^8^	4.89 × 10^8^	1.96 × 10^8^	6.36 × 10^7^	2.02 × 10^7^	6.38 × 10^6^
20	7.01 × 10^8^	6.89 × 10^8^	5.62 × 10^8^	2.30 × 10^8^	7.45 × 10^7^	2.36 × 10^7^	7.47 × 10^6^
30	6.77 × 10^8^	6.70 × 10^8^	5.71 × 10^8^	2.35 × 10^8^	7.59 × 10^7^	2.41 × 10^7^	7.61 × 10^6^
40	7.15 × 10^8^	7.09 × 10^8^	6.04 × 10^8^	2.39 × 10^8^	7.71 × 10^7^	2.44 × 10^7^	7.73 × 10^6^
50	7.17 × 10^8^	7.13 × 10^8^	6.11 × 10^8^	2.40 × 10^8^	7.74 × 10^7^	2.45 × 10^7^	7.76 × 10^6^
**DLG Zigzag (8, 8)**							
10	4.46 × 10^8^	4.35 × 10^8^	3.49 × 10^8^	1.54 × 10^8^	5.10 × 10^7^	1.62 × 10^7^	5.13 × 10^6^
20	4.55 × 10^8^	4.50 × 10^8^	3.96 × 10^8^	1.93 × 10^8^	6.42 × 10^7^	2.04 × 10^7^	6.46 × 10^6^
30	4.82 × 10^8^	4.78 × 10^8^	4.35 × 10^8^	2.12 × 10^8^	7.00 × 10^7^	2.22 × 10^7^	7.03 × 10^6^
40	4.88 × 10^8^	4.85 × 10^8^	4.50 × 10^8^	2.18 × 10^8^	7.14 × 10^7^	2.27 × 10^7^	7.17 × 10^6^
50	4.73 × 10^8^	4.71 × 10^8^	4.45 × 10^8^	2.17 × 10^8^	7.12 × 10^7^	2.26 × 10^7^	7.15 × 10^6^
**DLG Zigzag (10, 10)**							
10	3.13 × 10^8^	3.07 × 10^8^	2.56 × 10^8^	1.23 × 10^8^	4.12 × 10^7^	1.31 × 10^7^	4.15 × 10^6^
20	3.30 × 10^8^	3.27 × 10^8^	2.96 × 10^8^	1.62 × 10^8^	5.58 × 10^7^	1.78 × 10^7^	5.63 × 10^6^
30	3.51 × 10^8^	3.49 × 10^8^	3.26 × 10^8^	1.86 × 10^8^	6.35 × 10^7^	2.02 × 10^7^	6.40 × 10^6^
40	3.58 × 10^8^	3.57 × 10^8^	3.39 × 10^8^	1.96 × 10^8^	6.64 × 10^7^	2.11 × 10^7^	6.68 × 10^6^
50	3.51 × 10^8^	3.50 × 10^8^	3.37 × 10^8^	1.99 × 10^8^	6.69 × 10^7^	2.13 × 10^7^	6.73 × 10^6^
**DLG Armchair (12, 0)**							
10	4.45 × 10^8^	4.38 × 10^8^	3.81 × 10^8^	1.94 × 10^8^	6.62 × 10^7^	2.11 × 10^7^	6.68 × 10^6^
20	5.24 × 10^8^	5.17 × 10^8^	4.42 × 10^8^	2.02 × 10^8^	6.65 × 10^7^	2.11 × 10^7^	6.68 × 10^6^
30	5.35 × 10^8^	5.32 × 10^8^	4.81 × 10^8^	2.22 × 10^8^	7.25 × 10^7^	2.30 × 10^7^	7.27 × 10^6^
40	5.37 × 10^8^	5.35 × 10^8^	4.93 × 10^8^	2.25 × 10^8^	7.34 × 10^7^	2.33 × 10^7^	7.36 × 10^6^
50	5.39 × 10^8^	5.38 × 10^8^	5.05 × 10^8^	2.26 × 10^8^	7.36 × 10^7^	2.33 × 10^7^	7.38 × 10^6^
**DLG Armchair (16, 0)**							
10	3.37 × 10^8^	3.29 × 10^8^	2.69 × 10^8^	1.23 × 10^8^	4.11 × 10^7^	1.31 × 10^7^	4.14 × 10^6^
20	3.67 × 10^8^	3.63 × 10^8^	3.26 × 10^8^	1.71 × 10^8^	5.81 × 10^7^	1.85 × 10^7^	5.85 × 10^6^
30	3.69 × 10^8^	3.66 × 10^8^	3.42 × 10^8^	1.90 × 10^8^	6.45 × 10^7^	2.05 × 10^7^	6.50 × 10^6^
40	3.72 × 10^8^	3.70 × 10^8^	3.52 × 10^8^	2.00 × 10^8^	6.72 × 10^7^	2.14 × 10^7^	6.77 × 10^6^
50	3.75 × 10^8^	3.74 × 10^8^	3.63 × 10^8^	2.05 × 10^8^	6.83 × 10^7^	2.17 × 10^7^	6.86 × 10^6^
**DLG Armchair (20, 0)**							
10	2.98 × 10^8^	2.92 × 10^8^	2.43 × 10^8^	1.16 × 10^8^	3.90 × 10^7^	1.24 × 10^7^	3.93 × 10^6^
20	2.47 × 10^8^	2.44 × 10^8^	2.25 × 10^8^	1.34 × 10^8^	4.74 × 10^7^	1.52 × 10^7^	4.80 × 10^6^
30	2.69 × 10^8^	2.68 × 10^8^	2.54 × 10^8^	1.61 × 10^8^	5.72 × 10^7^	1.83 × 10^7^	5.79 × 10^6^
40	2.82 × 10^8^	2.81 × 10^8^	2.70 × 10^8^	1.77 × 10^8^	6.23 × 10^7^	1.99 × 10^7^	6.30 × 10^6^
50	2.78 × 10^8^	2.77 × 10^8^	2.71 × 10^8^	1.82 × 10^8^	6.37 × 10^7^	2.03 × 10^7^	6.42 × 10^6^

**Table 4:** Cantilever SLG zigzag and armchair with mass addition at tip of sheet.

Length of Sheet (Nm)	Mass (gm)						
	1.00 × 10^−22^	1.00 × 10^−21^	1.00 × 10^−20^	1.00 × 10^−19^	1.00 × 10^−18^	1.00 × 10^−17^	1.00 × 10^−16^
**DLG Zigzag (6,6)**							
10	1.43 × 10^7^	1.06 × 10^7^	4.53 × 10^6^	1.49 × 10^6^	4.74 × 10^5^	1.50 × 10^5^	1.00 × 10^5^
20	1.51 × 10^7^	1.37 × 10^7^	8.12 × 10^6^	2.96 × 10^6^	9.50 × 10^5^	3.01 × 10^5^	1.01 × 10^5^
30	1.51 × 10^7^	1.41 × 10^7^	9.19 × 10^6^	3.51 × 10^6^	1.14 × 10^6^	3.60 × 10^5^	1.14 × 10^5^
40	1.52 × 10^7^	1.44 × 10^7^	9.97 × 10^6^	3.94 × 10^6^	1.28 × 10^6^	4.06 × 10^5^	1.28 × 10^5^
50	1.63 × 10^7^	1.56 × 10^7^	1.14 × 10^7^	4.64 × 10^6^	1.52 × 10^6^	4.81 × 10^5^	1.52 × 10^5^
**DLG Zigzag (8,8)**							
10	8.13 × 10^6^	7.11 × 10^6^	3.86 × 10^6^	1.36 × 10^6^	4.35 × 10^5^	1.38 × 10^5^	1.00 × 10^5^
20	8.24 × 10^6^	7.65 × 10^6^	4.92 × 10^6^	1.87 × 10^6^	6.05 × 10^5^	1.92 × 10^5^	1.01 × 10^5^
30	8.33 × 10^6^	7.90 × 10^6^	5.57 × 10^6^	2.25 × 10^6^	7.35 × 10^5^	2.33 × 10^5^	2.00 × 10^5^
40	8.44 × 10^6^	8.10 × 10^6^	6.03 × 10^6^	2.55 × 10^6^	8.41 × 10^5^	2.67 × 10^5^	1.12 × 10^5^
50	1.13 × 10^7^	1.09 × 10^7^	8.10 × 10^6^	3.39 × 10^6^	1.11 × 10^6^	3.53 × 10^5^	1.12 × 10^5^
**DLG Zigzag (10,10)**							
10	5.18 × 10^6^	4.63 × 10^6^	2.66 × 10^6^	9.58 × 10^5^	3.07 × 10^5^	1.00 × 10^5^	1.00 × 10^5^
20	5.21 × 10^6^	4.90 × 10^6^	3.34 × 10^6^	1.32 × 10^6^	4.29 × 10^5^	1.36 × 10^5^	1.01 × 10^5^
30	5.21 × 10^6^	5.00 × 10^6^	3.71 × 10^6^	1.58 × 10^6^	5.20 × 10^5^	1.65 × 10^5^	1.03 × 10^5^
40	5.21 × 10^6^	5.05 × 10^6^	4.00 × 10^6^	1.83 × 10^6^	6.11 × 10^5^	1.94 × 10^5^	1.12 × 10^5^
50	5.20 × 10^6^	5.07 × 10^6^	4.17 × 10^6^	1.99 × 10^6^	6.74 × 10^5^	2.15 × 10^5^	1.13 × 10^5^
**DLG Armchair (12,0)**							
10	1.07 × 10^7^	9.15 × 10^6^	4.76 × 10^6^	1.65 × 10^6^	5.27 × 10^5^	1.67 × 10^5^	1.00 × 10^5^
20	1.13 × 10^7^	1.04 × 10^7^	6.50 × 10^6^	2.44 × 10^6^	7.87 × 10^5^	2.50 × 10^5^	1.69 × 10^5^
30	1.13 × 10^7^	1.07 × 10^7^	7.43 × 10^6^	2.97 × 10^6^	9.06 × 10^5^	2.87 × 10^5^	1.10 × 10^5^
40	1.12 × 10^7^	1.07 × 10^7^	7.62 × 10^6^	3.10 × 10^6^	1.01 × 10^6^	3.21 × 10^5^	1.03 × 10^5^
50	1.13 × 10^7^	1.09 × 10^7^	8.10 × 10^6^	3.38 × 10^6^	1.11 × 10^6^	3.52 × 10^5^	1.11 × 10^5^
**DLG Armchair (16,0)**							
10	5.93 × 10^6^	5.24 × 10^6^	2.91 × 10^6^	1.03 × 10^6^	3.31 × 10^5^	1.05 × 10^5^	1.00 × 10^5^
20	6.23 × 10^6^	5.84 × 10^6^	3.91 × 10^6^	1.53 × 10^6^	4.95 × 10^5^	1.57 × 10^5^	1.01 × 10^5^
30	6.24 × 10^6^	5.95 × 10^6^	4.31 × 10^6^	1.78 × 10^6^	5.84 × 10^5^	1.85 × 10^5^	1.03 × 10^5^
40	6.19 × 10^6^	5.97 × 10^6^	4.56 × 10^6^	1.98 × 10^6^	6.57 × 10^5^	2.09 × 10^5^	1.12 × 10^5^
50	6.26 × 10^6^	6.08 × 10^6^	4.82 × 10^6^	2.19 × 10^6^	7.31 × 10^5^	2.32 × 10^5^	1.13 × 10^5^
**DLG Armchair (20,0)**							
10	3.56 × 10^6^	3.20 × 10^6^	1.85 × 10^6^	6.69 × 10^5^	2.15 × 10^5^	1.19 × 10^5^	1.00 × 10^5^
20	3.75 × 10^6^	3.54 × 10^6^	2.46 × 10^6^	9.86 × 10^5^	3.22 × 10^5^	1.02 × 10^5^	1.00 × 10^5^
30	3.76 × 10^6^	3.62 × 10^6^	2.76 × 10^6^	1.21 × 10^6^	3.99 × 10^5^	1.27 × 10^5^	1.03 × 10^5^
40	3.73 × 10^6^	3.62 × 10^6^	2.90 × 10^6^	1.34 × 10^6^	4.50 × 10^5^	1.43 × 10^5^	1.12 × 10^5^
50	3.77 × 10^6^	3.69 × 10^6^	3.09 × 10^6^	1.54 × 10^6^	5.25 × 10^5^	1.67 × 10^5^	1.19 × 10^5^

**Table 5:** Bridged SLG zigzag and armchair with mass addition at centre of sheet.

Length of Sheet (Nm)	Mass (gm)						
	1.00 × 10^−22^	1.00 × 10^−21^	1.00 × 10^−20^	1.00 × 10^−19^	1.00 × 10^−18^	1.00 × 10^−17^	1.00 × 10^−16^
**DLG Zigzag (6, 6)**							
10	9.59 × 10^7^	8.49 × 10^7^	4.70 × 10^7^	1.66 × 10^7^	5.31 × 10^6^	1.68 × 10^6^	5.32 × 10^5^
20	9.86 × 10^7^	9.20 × 10^7^	5.76 × 10^7^	2.10 × 10^7^	6.74 × 10^6^	2.14 × 10^6^	6.75 × 10^5^
30	9.88 × 10^7^	9.41 × 10^7^	6.09 × 10^7^	2.21 × 10^7^	7.10 × 10^6^	2.25 × 10^6^	7.11 × 10^5^
40	9.90 × 10^7^	9.54 × 10^7^	6.25 × 10^7^	2.27 × 10^7^	7.27 × 10^6^	2.30 × 10^6^	7.28 × 10^5^
50	1.07 × 10^8^	1.03 × 10^8^	6.51 × 10^7^	2.34 × 10^7^	7.49 × 10^6^	2.37 × 10^6^	7.50 × 10^5^
**DLG Zigzag (8, 8)**							
10	3.54 × 10^7^	3.27 × 10^7^	2.07 × 10^7^	7.81 × 10^6^	2.52 × 10^6^	7.99 × 10^5^	2.53 × 10^5^
20	7.38 × 10^6^	7.33 × 10^6^	6.83 × 10^6^	4.38 × 10^6^	1.64 × 10^6^	5.29 × 10^5^	1.68 × 10^5^
30	5.45 × 10^7^	5.26 × 10^7^	3.88 × 10^7^	1.56 × 10^7^	5.06 × 10^6^	1.60 × 10^6^	5.07 × 10^5^
40	5.51 × 10^7^	5.36 × 10^7^	4.02 × 10^7^	1.61 × 10^7^	5.23 × 10^6^	1.66 × 10^6^	5.24 × 10^5^
50	7.33 × 10^7^	7.14 × 10^7^	5.11 × 10^7^	1.94 × 10^7^	6.25 × 10^6^	1.98 × 10^6^	6.26 × 10^5^
**DLG Zigzag (10, 10)**							
10	3.40 × 10^7^	3.15 × 10^7^	2.01 × 10^7^	7.58 × 10^6^	2.45 × 10^6^	7.77 × 10^5^	2.46 × 10^5^
20	3.42 × 10^7^	3.29 × 10^7^	2.43 × 10^7^	1.02 × 10^7^	3.33 × 10^6^	1.06 × 10^6^	3.35 × 10^5^
30	3.56 × 10^7^	3.46 × 10^7^	2.73 × 10^7^	1.19 × 10^7^	3.91 × 10^6^	1.24 × 10^6^	3.93 × 10^5^
40	3.43 × 10^7^	3.36 × 10^7^	2.77 × 10^7^	1.25 × 10^7^	4.12 × 10^6^	1.31 × 10^6^	4.14 × 10^5^
50	3.43 × 10^7^	3.38 × 10^7^	2.84 × 10^7^	1.28 × 10^7^	4.21 × 10^−01^	1.34 × 10^6^	4.23 × 10^5^
**DLG Armchair (12, 0)**							
10	6.95 × 10^7^	6.17 × 10^7^	3.45 × 10^7^	1.23 × 10^7^	3.45 × 10^7^	1.24 × 10^6^	3.93 × 10^5^
20	7.29 × 10^7^	6.85 × 10^7^	4.51 × 10^7^	1.70 × 10^7^	5.49 × 10^6^	1.74 × 10^6^	5.50 × 10^5^
30	7.31 × 10^7^	7.01 × 10^7^	4.86 × 10^7^	1.85 × 10^7^	5.97 × 10^6^	1.89 × 10^6^	5.99 × 10^5^
40	7.26 × 10^7^	7.03 × 10^7^	4.98 × 10^7^	6.10 × 10^6^	1.93 × 10^6^	1.93 × 10^6^	6.11 × 10^5^
50	7.33 × 10^7^	7.12 × 10^7^	5.04 × 10^7^	1.90 × 10^7^	6.14 × 10^6^	1.94 × 10^6^	6.15 × 10^5^
**DLG Armchair (16, 0)**							
10	3.84 × 10^7^	3.50 × 10^7^	2.12 × 10^7^	7.81 × 10^6^	2.52 × 10^6^	7.97 × 10^5^	2.52 × 10^5^
20	4.02 × 10^7^	3.83 × 10^7^	2.76 × 10^7^	1.12 × 10^7^	3.66 × 10^6^	1.16 × 10^6^	3.67 × 10^5^
30	4.03 × 10^7^	3.90 × 10^7^	3.01 × 10^7^	1.28 × 10^7^	4.19 × 10^6^	1.33 × 10^6^	4.21 × 10^5^
40	3.99 × 10^7^	3.91 × 10^7^	3.14 × 10^7^	1.35 × 10^7^	4.44 × 10^6^	1.41 × 10^6^	4.46 × 10^5^
50	4.03 × 10^7^	3.96 × 10^7^	3.22 × 10^7^	1.38 × 10^7^	4.53 × 10^6^	1.44 × 10^6^	4.55 × 10^5^
**DLG Armchair (20, 0)**							
10	2.31 × 10^7^	2.15 × 10^7^	1.41 × 10^7^	5.38 × 10^6^	1.74 × 10^6^	5.52 × 10^5^	1.75 × 10^5^
20	2.41 × 10^7^	2.33 × 10^7^	1.77 × 10^7^	7.67 × 10^6^	2.54 × 10^6^	8.06 × 10^5^	2.55 × 10^5^
30	2.42 × 10^7^	2.36 × 10^7^	1.93 × 10^7^	8.99 × 10^6^	3.01 × 10^6^	9.58 × 10^5^	3.03 × 10^5^
40	2.40 × 10^7^	2.36 × 10^7^	2.00 × 10^7^	9.66 × 10^6^	3.25 × 10^6^	1.03 × 10^6^	3.27 × 10^5^
50	2.42 × 10^7^	2.39 × 10^7^	2.07 × 10^7^	1.02 × 10^7^	3.42 × 10^6^	1.09 × 10^6^	3.44 × 10^5^
